# Brain asymmetry is globally different in males and females: exploring cortical volume, area, thickness, and mean curvature

**DOI:** 10.1093/cercor/bhad396

**Published:** 2023-10-17

**Authors:** Magda L Dumitru

**Affiliations:** Department of Biological Sciences, University of Bergen, Postboks 7803, 5020 Bergen, Norway; Department of Biological and Medical Psychology, University of Bergen, Postboks 7807, 5020 Bergen, Norway

**Keywords:** brain asymmetry, coherence, distance index, laterality, sexual dimorphism

## Abstract

Brain asymmetry is a cornerstone in the development of higher-level cognition, but it is unclear whether and how it differs in males and females. Asymmetry has been investigated using the laterality index, which compares homologous regions as pairwise weighted differences between the left and the right hemisphere. However, if asymmetry differences between males and females are global instead of pairwise, involving proportions between multiple brain areas, novel methodological tools are needed to evaluate them. Here, we used the Amsterdam Open MRI collection to investigate sexual dimorphism in brain asymmetry by comparing laterality index with the distance index, which is a global measure of differences within and across hemispheres, and with the subtraction index, which compares pairwise raw values in the left and right hemisphere. Machine learning models, robustness tests, and group analyses of cortical volume, area, thickness, and mean curvature revealed that, of the three indices, distance index was the most successful biomarker of sexual dimorphism. These findings suggest that left–right asymmetry in males and females involves global coherence rather than pairwise contrasts. Further studies are needed to investigate the biological basis of local and global asymmetry based on growth patterns under genetic, hormonal, and environmental factors.

## Introduction

Brain asymmetry develops before birth (Rajagopalan et al. 2011), with specific range and distribution patterns across males and females still a matter of debate (Gilmore et al. 2007; Guo et al. 2016; Lehtola et al. 2019; Ruigrok et al. 2014). Overall, a series of growth mechanisms are in place (Debat and Peronnet 2013; Lander 2011; Rao et al. 2002; Waddington 1959) for achieving a specific “target size” for body and its component parts (Tanner 1963), including catch up growth in children after malnutrition or sickness (Boersma and Wit 1997; Prader, Tanner, von Harnack 1963; Tanner 1963). Moreover, the size of bilateral body parts such as arms or legs is subject to contralateral regulation (for a review of current debates, see Genikhovich and Technau 2017), such that growth on one side of the body would signal growth on the other side (Roselló-Díez et al. 2018). In sum, both systemic growth retardation and direct left–right communication are driving body symmetry (Busse et al. 2018; Fischerauer et al. 2013) by overriding genetic and environmental variation (Grimes 2019). Nevertheless, certain asymmetries survive, for example at the brain level, including intra-hemispheric gray matter volume (Esteves et al. 2019), cortical thickness (Kong et al. 2018), surface area, and gyrification (Chiarello et al. 2016). The main role of these asymmetries would be to support high-level cognition including episodic memory (Habib et al. 2003), emotion identification (Brunoni et al. 2016), face processing (Zhen et al. 2015), and language (de Courten-Myers 1999; Good et al. 2001; Shapleske et al. 1999).

Whether brain asymmetries are distinct in males and females is far from clear. Moreover, it is unclear what type of asymmetries are specific to (young) adult male and female brains, and current evidence based on weighted pairwise differences remains inconsistent. For example, measurements for the “planum temporale,” which subserves language, sometimes supports stronger leftward lateralization in males (Guadalupe et al. 2015), and sometimes stronger leftward lateralization in females (Ruigrok et al. 2014). Overall, few, if any, significant differences are left standing between sexes after controlling for total intracranial volume (cf. review by Sanchis-Segura et al. 2019). Current methods for computing brain asymmetry may be responsible for the stalemate, as they fail to capture potentially different types of structural differences. The “laterality index” (LI) helps determine, for instance, whether the left planum temporale is larger than the right, without considering the impact of other brain areas or underlying growth differences of brain structures in males and females.

Here, we investigated sexual dimorphism in brain asymmetry by comparing the performance of three structural indices measuring distinct qualitative differences between the left and the right hemisphere. We used the Amsterdam Open MRI collection (Snoek et al. 2020) to compute absolute values of the pairwise laterality index for each subject and for each of the 34 regions of interest (ROIs) in the Desikan–Tourville atlas (Desikan et al. 2006). Second, we computed a “subtraction index” (SI) as absolute values of raw pairwise differences between left and the right regions for each of the 34 ROIs. Third, we computed a global “distance index” (DI) by correlating two series of differences—between each ROI and all other ROIs in the left hemisphere, and between each homologous ROI and all other ROIs in the right hemisphere, as described under “Materials and Methods”. We repeated the computation of DI, LI, and SI for several gray matter measures, namely cortical volume, area, thickness, and mean curvature, and evaluated their performance using machine learning and robustness analyses drawing on image-quality metrics.

Each index captures specific growth patterns that may depend on proportion, scaling, and/or size regulation of the two hemispheres and their constituent parts. Therefore, an investigation of their capacity to distinguish males from females would help identify which brain growth patterns are ecologically valid. Indeed, the rules governing form and proportion are fundamental to the evolution and development of biological organisms and account for body size growth, interaction among specific tissues, and inherent positional identity of constituent parts (Harris et al. 2021). We therefore expect these rules to also be relevant to hemispheric asymmetry patterns in humans, which remain un(der)explored. Several hypotheses can be put forward on which asymmetry indices could successfully capture sexual dimorphism in cortical asymmetry. First, the laterality index, which relies on weighted pairwise differences, might outperform DI and SI if the 34 brain regions had similar growth rates and proportions across hemispheres in males and females, but with decreased scaling in females, which would require activation of coordinated scaling of growth hormone, growth factors, and thyroid hormone (Harris et al. 2021). Second, the subtraction index, which relies on overall size differences, might outperform DI and LI if the 34 brain regions involved differences in target size across hemispheres for males and females, whether or not regions maintain similar proportions to the right and to the left, as long as metrics are overall different across the sexes (e.g. higher values for males compared with females). Third, the distance index, which relies on global coherence within and across hemispheres, might outperform LI and SI if it were sensitive to hemisphere target size, to interactions among ROIs within each hemisphere, as well as to inherent positional identity of each region, such that effective signaling between ipsilateral and contralateral regions could occur.

A certain overlap in performance can be expected among the three indices, as they all involve differences in asymmetry magnitude between males and females. However, we do not expect conflicting results for sexual dimorphism among indices or cortical measures such that higher metrics are elicited for males, for instance, under random combinations of index and measures, which would question the reliability of (some) indices to capture differences between males and females. Details on how each index were computed as well as on further analyses are presented below.

## Materials and methods

### Participants

A total of 928 healthy participants are included in the freely available ID1000 dataset of the Amsterdam Open MRI collection (https://openneuro.org/datasets/ds003097) following standard ethics procedures. We analyzed structural (T1-weighted) scans for a subset of 826 right-handed individuals (396 males and 430 females), for whom detailed information is available from Snoek and colleagues (https://doi.org/10.1101/2020.06.16.155317).

### MRI data acquisition and preprocessing

Neuroimaging data were acquired on a 3 T Philips Intera scanner (Philips, Best, the Netherlands). T1-weighted high-resolution anatomical scans (3D MPRAGE) were collected with the following parameters: 1 × 1 × 1 voxel size, TR/TE = 8.1/3.7 ms, FOV = 160 × 256 × 256 mm^3^, Flip angle = 8°. Cortical reconstruction, volumetric segmentation, and parcellation using the Desikan-Killiany atlas (Desikan et al. 2006) were performed with the *Freesurfer 6.0.1* image analysis suite, which is documented (Dale, Fischl, and Sereno, 1999) and can be download online (http://surfer.nmr.mgh.harvard.edu/). Brain surface was reconstructed using the same version of *FreeSurver* and brain mask estimated by reconciling ANTs and FreeSurfer segmentations of cortical gray-matter specific to *Mindboggle* (Klein et al. 2017). Spatial normalization to the ICBM 152 Nonlinear Asymmetrical template version 2009c (Fonov et al. 2009) was performed using nonlinear registration with *ANTs v2.1.0* (Avants et al. 2008) and using T1w volume as well as template. T1w segmentation was done using fast *FSL v5.0.9* (Zhang, Brady, and Smith 2001)..

### Asymmetry indices

We computed three asymmetry indices using Matlab 2022a (Mathworks 2022) based on raw values for 34 regions following the Desikan–Tourville atlas (Desikan et al. 2006) across several gray matter measures: volume, surface area, thickness, and mean curvature (henceforth “curvature”). We derived the distance index as detailed in equation (1). To obtain a DI value for one region (DI_i_), we first computed a vector of absolute differences between that region in the left hemisphere and the other regions ipsilaterally, and then a vector of absolute differences between the same region contralaterally and the other regions in the right hemisphere. After correlating the two vectors, we computed a Pearson correlation coefficient, which we subtracted from 1 to derive DI as the absolute value for that ROI. The higher the DI, the greater the asymmetry (i.e. difference in magnitude between hemispheres) for that region. Conversely, the lower the DI, the smaller the cortical asymmetry. By repeating the computation, we obtained a 34-dimension vector that we compared across two groups of participants (e.g. males and females) in a paired-sample *t*-test:


(1) \begin{equation*} {DI}_i=1-\left|\frac{\sum \left({a}_{i, Left}-\overline{Vector_{Left}}\right)\ \left({a}_{i, Right}-\overline{Vector_{Right}}\right)}{\sqrt{\sum{\left({a}_{i, Left}-\overline{Vector_{Left}}\right)}^2\sum{\left({a}_{i, Right}-\overline{Vector_{Right}}\right)}^2}}\right| \end{equation*}

where


$${Vector}_{Left}=\left[\left|{a}_{i, Left}-{a}_{i+1, Left}\right|\kern0.5em \cdots \kern0.5em \left|{a}_{i, Left}-{a}_{n, Left}\right|\right]$$

$${Vector}_{Right}=\left[\begin{array}{ccc}\left|{a}_{i, Right}-{a}_{i+1, Right}\right|& \cdots & \left|{a}_{i, Right}-{a}_{n, Right}\right|\end{array}\right]$$

For the laterality index for a particular region (LI_i_), we computed a modified formula of the classic laterality index, as detailed in equation (2), by subtracting the value of that region in the left hemisphere from its counterpart in the right hemisphere before dividing this number by the sum of the two. In a last step, we derived absolute values to obtain the LI for each region. In doing so, we estimated the magnitude of the left–right asymmetry rather than the sign of this difference, as we were not interested which hemisphere was larger than the other. We obtained a series of positive differences, one per ROI, which we further compared between males and females:


(2) \begin{equation*} {LI}_i=\left|\frac{a_{i, Left}-{a}_{i, Right}}{a_{i, Left}+{a}_{i, Right}}\right| \end{equation*}

To compute the subtraction index for a particular region (SI_i_), we derived the difference between the raw value of that region and their counterpart in the contralateral hemisphere before deriving the absolute value of this difference, as detailed in equation (3). Again, we aimed to measure the magnitude of the left–right difference instead of the sign of a particular value. Importantly, unlike DI, both LI and SI derive pairwise differences for each region and do not factor in other regions when estimating the asymmetry of a single region. The distinction to be made between LI and SI is that only the former involves proportional/weighted left–right differences, whereas the latter simply measures raw left–right differences. In sum, the three indices provide asymmetry estimates for homologous ROIs across hemispheres in the form of positive differences in magnitude, to be compared between males and females:


(3) \begin{equation*} {SI}_i=\left|{a}_{i, Left}-{a}_{i, Right}\right| \end{equation*}

### Binary classification models

All statistical analyses were carried out in the statistical environment *R*, version 2022 July 2 (R Core Team 2012) using version 6.0–94 of the Classification and Regression Training (“caret”) package for machine learning (Kuhn 2008). We set a unique random seed before building models for each index and for each cortical measure, then split the data into a training set (80%) and a test set (20%). A Random Forest classifier (Breiman 2001) was run for each index and measure including values for all 34 ROIs plus age as predictors. Random Forest is a popular supervised machine learning algorithm that generates and combines multiple decision trees that are trained on different parts of a given set, thereby providing clear advantages over other machine learning methods, such as optimized prediction accuracy and overfitting. Reliability is built into Random Forest models as “bagging,” which involves aggregation and bootstrapping. To further enhance reliability, we applied 10-fold grid leave-one-out cross-validation over test data.

A confusion matrix and associated statistics (i.e. model accuracy, *Cohen’s Kappa*, and *P*-values) was computed for each index and for each cortical measure (i.e. area, volume, thickness, and curvature) to better evaluate the algorithms. A confusion matrix was derived for each model as a 2 × 2 table that summarizes the percentage of correctly and incorrectly classified males and females. The “Cross-Validation for Model Selection” (cvms) package in R (Jeyaraman et al. 2019, https://cran.r-project.org/package=cvms) was used to visualize the confusion matrices. Model accuracy specifies how many correct predictions (i.e. true positives and true negatives) the classification model will make over 100 participants. However, even when accuracy is high, it must be interpreted together with *Cohen’s Kappa*, which corrects for the likelihood that accuracy values might occur accidentally by taking into account both true positives and false positives. The rule of thumb is that *Kappa* coefficients lower than zero indicate worse-than-chance classification performance, over 0.20 fair performance, over 0.40 good performance, and over 0.60 up to 1 very good classification performance (Fleiss 1981). However, interpreting *Kappa* values is highly dependent on the research question and may be inadequate when marginals are similar for predicted and target groups (Delgado and Tibau 2019). Accuracy values must also exceed the “no information rate” (NIR), which is the accuracy obtained by always predicting the majority class. The model should thus yield a significant *p*-value between accuracy and NIR. Further, Random Forest allows us to estimate the relative importance of model predictors that is, the 34 cortical regions plus age. We plotted the six highest ranking predictors on cortical surface maps (cf. Mowinckel and Vidal-Pineiro 2019) for each index and for each cortical measure.

### Robustness and reliability analyses

The quality of magnetic resonance (MR) images may depend on operator errors, patient movement, equipment performance, as well as by contrast and brightness as indexed by T1-weighted (T1w) sequences. Image-quality metrics are typically not included in brain imaging studies, but failure to control for data quality was shown to bias estimates of lifespan brain development (Rosen et al. 2018; Zuo et al. 2017) and age (Power et al. 2012; Roalf et al. 2016). Evidence of negligible impact of image-quality metrics on index values would support index robustness. Indeed, analyses are robust when they do not vary strongly with external parameters (e.g. image quality), and they are reliable when they do not vary across repeated testing of the same sample (Jansen et al. 2006). In our study, reliability is part of the Random Forest algorithm, which involves bootstrapping as well as cross validation. In addition, obtaining similar result patterns for indices across several cortical measures, namely volume, area, thickness, and mean curvature, would further support index reliability.

The AOMIC database provides five of the 14 fully automated image-quality metrics described by Esteban and colleagues (Esteban et al. 2017). The five metrics are based on specific artifacts, structural image quality, and information theory. Measures targeting specific artifacts are the intensity nonuniformity metric (INU) and the white matter to maximum intensity ration (WM2MAX). The former describes the inhomogeneity of the magnetic field, with values around 1.0 indicating less motion and smaller bias in the magnetic field. The latter measures median intensity of white matter over 95% of full intensity distribution and captures artifacts generated by carotid vessels and fat. Values between 0.6 and 0.8 indicate ideal white matter brightness. Structural image quality measures evaluate the impact of noise and include the coefficient of joint variation (CJV) of white and gray matter and the contrast-to-noise ratio (CNR). The former is an objective function for optimizing INU correction algorithms (Ganzetti et al. 2016a), with higher values indicating head motion and important INU artifacts. The latter evaluates the degree of separation between gray matter and white matter, with higher values indicating better tissue separation. Finally, information theory measures evaluate the spatial distribution of information. In particular, the entropy-focus criterion (EFC) estimates ghosting and blurring caused by head motion; hence, lower values are preferable (Atkinson et al. 1997).

To evaluate the impact of image-quality metrics on the robustness of the three asymmetry indices, we selected two equal subsamples for each metric and for each group, as follows. First, data were organized in lower and upper levels for each image-quality metric, and 80 participants (approx. 20%) were selected for each level, while matching the metrics values across males and females. As a rule, the first and the last 80 participants and corresponding index values were selected for each metric. EFC was an exception, as values were highly homogeneous in females compared with males, and therefore upper and lower groups could only be delineated by strongly polarizing the samples. As a result, two groups of only 35 participants each were selected. In a last step, paired-sample *t*-tests were run between males and females index values across the 34 ROIs for each metric and for each level. Several outcomes are possible, bearing in mind that some image-quality metrics are better when higher (e.g. CNR, INU), whereas others are better when lower (i.e. CJV, EFC). First, differences between males and females could be significant only for the preferred level, which would indicate that a particular index, either DI, LI, or SI, is sensitive to image quality metrics. Second, analyses of asymmetry indices for males and females could be significant for both upper and lower metrics levels, in which case the index is a robust biomarker of sexual dimorphism. Third, there may be no significant differences for a given index between males and females for associated lower or upper image-metrics, which would indicate that the index is not a robust biomarker or brain asymmetry.

### Group analyses and correlations with males/females

Group analyses as paired *t*-tests between males and females for each index and for each cortical measure were performed, whose results could support the output of binary classification models. Pearson correlations between sex and whole-brain index values were computed, allowing for an informal comparison of asymmetry indices and their potential to distinguish males from females across cortical measures. For each ROI, we computed index values (separately for DI, LI, and SI) across subjects and correlated them with males/females (coded as “0” and “1,” respectively) before averaging over correlation coefficients for that particular region and for each index. In a last step, we obtained a grand average over all 34 ROI for each index and represented the three values in graphical format. Another informal analysis was performed over raw ROI values for each hemisphere, to determine the potential of each hemisphere alone to provide cues for binary classification. As before, correlations with males/females were averaged for each ROI and for each hemisphere, followed by a grand average computed for each hemisphere and cortical measure before representing values in graphical format.

## Results

### Random Forest binary classification

**Fig. 1:**
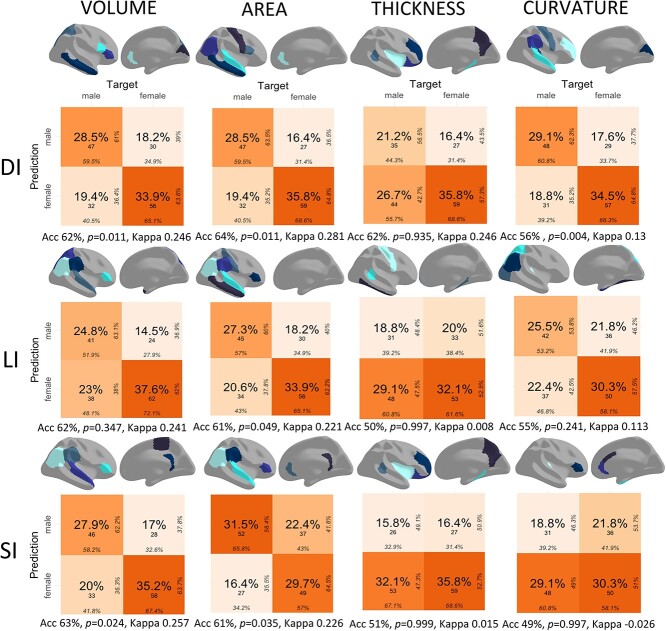
Performance of binary classification models. Each 2 × 2 confusion matrix corresponds to a specific measure (volume, area, thickness, or curvature) and index (DI, LI, or SI) with accuracy, *p*-value relative to no-information rate, and *Cohen's Kappa* given underneath. True positive, false positive, false negative, and true negative quadrants in each matrix include overall percentage and count in the middle. Column percentages are given at the bottom and row percentages to the right in each quadrant. Six regions of interest are displayed on lateral and medial cortical maps, ranking from most important (lightest) to least important (darkest) for each model.

The trained model was tested on the test dataset to obtain single-subject predictions in the form of accuracy percentages and counts of correct and incorrect classification. Figure 1 illustrates a series of 2 × 2 confusion matrices summarizing the outcomes of Random Forest models, one for each index and cortical measure. The following associated statistics are reported underneath each matrix: accuracy (“*Acc”*), *P*-value when comparing accuracy with no-information rate, and *Cohen’s Kappa*. By combining target (raw) values over two columns and predicted values over two rows, four quadrants are defined, as follows: correct classification over the upper left and lower right quadrants (true positives for cases where males are predicted to be males and true negatives for cases where females are predicted to be females) and incorrect classification over the upper right and lower left quadrants (false positives in cases where females are predicted to be males and false negatives in cases where males are predicted to be females). Each quadrant in a matrix includes several numbers, starting with the overall percentage in the middle and overall count underneath. Column percentages are given at the bottom of each quadrant: if we consider the first matrix summarizing binary classifications for DI volume, of all the observations where Target is male, 59.5% were predicted to be male and 40.5% were predicted to be female. Row percentages are given to the right of the quadrants: if we consider the upper quadrants, of all the observations where Prediction is male, 61% of them were actually male, while 39% were female.

**Fig. 2:**
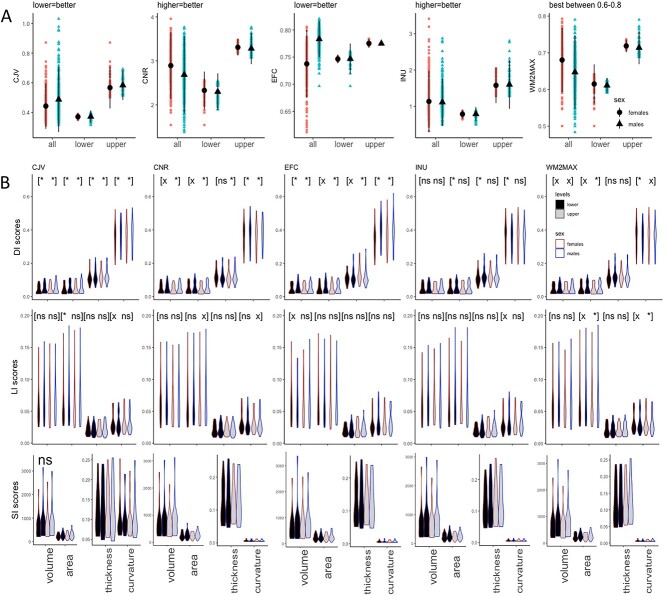
Image-quality metrics and their impact on asymmetry indices DI, LI, and SI. Average metric values (CJV, CNR, EFC, INU, and WM2MAX) are given for males and females in the full sample (all 826 right-handed participants) and in two subsamples corresponding to upper and lower metric levels (A). Average index values corresponding to upper and lower image-quality levels are given for males and females for cortical measures—volume, area, thickness, and curvature (B). Significance levels of *t*-tests between males and females are marked as “^*^” if less than 0.001, as “x” if less than 0.05 but higher than 0.001, and as “ns” if higher than 0.05. Note that SI scores are all “ns.”

The color intensity of the quadrants corresponds to counts, from deeper oranges (higher counts) to pale oranges (lower counts). Highly accurate classifications are therefore cases where the upper left—lower right diagonal is intensely colored, which was true for DI volume, area, and curvature, for LI area and curvature, and for SI volume and area. Accuracy levels exceeded 50% except for SI curvature, with DI scoring the highest for area, thickness, and curvature. *Cohen’s Kappa* values were above 20% only for DI volume area, and thickness, for LI volume and area, and for SI volume and area. As for *p*-values, they were significant for DI volume, area, and curvature, for LI area, and for SI volume and area, indicating that LI underperformed compared with the other two indices. DI curvature also featured reasonable statistics (56% accuracy and *P* = 0.004, despite a lower *Kappa* value of 0.13), whereas binary classifications based on cortical thickness fared poorly across indices.

Brain maps of lateral and medial surfaces above each confusion matrix in Fig. 1 present the six ROIs whose variance contributed to binary classification the most. Color intensity goes from light blue (highest rank) to dark blue (lowest rank). If we exclude thickness, for which classification was poor, we may conclude that successful classification relies mostly on prefrontal and temporal areas, as well as on the temporo-parietal junction, with slight variations across indices. Of the ROIs with highest differences in DI asymmetry between males and females, we singled out the rostral anterior cingulate for volume and area and the rostral middle frontal for mean curvature. For LI and SI, the inferior parietal ranked highly for volume and area, and even for mean curvature for LI. These areas are among those previously associated with male–female differences (i.e. Gomez et al. 2019; Lotze et al. 2019; Sowell et al. 2007), but their relevance to brain asymmetry was not established.

An important goal of the AOMIC database was to provide a homogeneous sample size and, therefore, only individuals in their early twenties were recruited. This goal was met, as we included “age” as a predictor of sex, alongside the 34 ROIs but found it among the lowest ranked predictors in the output of all models.

### Robustness analysis: Impact of image quality metrics on index values

Figure 2(A) summarizes five image-quality metrics (CJV, CNR, EFC, INU, and WM2MAX) associated with the 826 right-handed male and female participants in the AOMIC dataset. Overall, image quality was similar or even better compared with other large datasets, for example ABIDE (Di Martino et al. 2014). However, the values were different in males and females, which could bias the analysis of associated indices. To preclude biases when evaluating the impact of image quality metrics on index values, two subsamples were selected for each index and for each measure, corresponding with upper and lower image-quality levels. For each set of 34 ROIs at upper and lower levels, paired sample *t*-tests were run between males and females. Associated *p*-values were designated in Fig. 2(C) with “^*^” if less than 0.001, with “x” if less than 0.05 but higher than 0.001, and with “ns” if higher than 0.05.

Distance index scores were significantly higher in males compared with females for each cortical measure (volume, area, thickness, and curvature) across both lower and upper CJV and EFC, across lower and upper CNR and WM2MAX except for thickness, where differences were significant only for lower CNR, and finally for lower INU except volume. The results of *t*-test between males and females for the laterality index were significant only for a quarter of all *t*-tests run, namely for lower CJV area and curvature, upper CNR area and curvature, lower EFC volume, lower INU curvature, and upper WM2MAX area and curvature. Overall, asymmetry was again higher in males compared with females. Unlike DI scores, LI scores followed the optimal level of image-quality that is, they were significant if associated with lower CJV and EFC, as well as with higher CNR and INU. As for the subtraction index, scores were not significantly different for males and females across the board, suggesting low robustness for this index.

### Group analyses and correlations with males/females

**Fig. 3:**
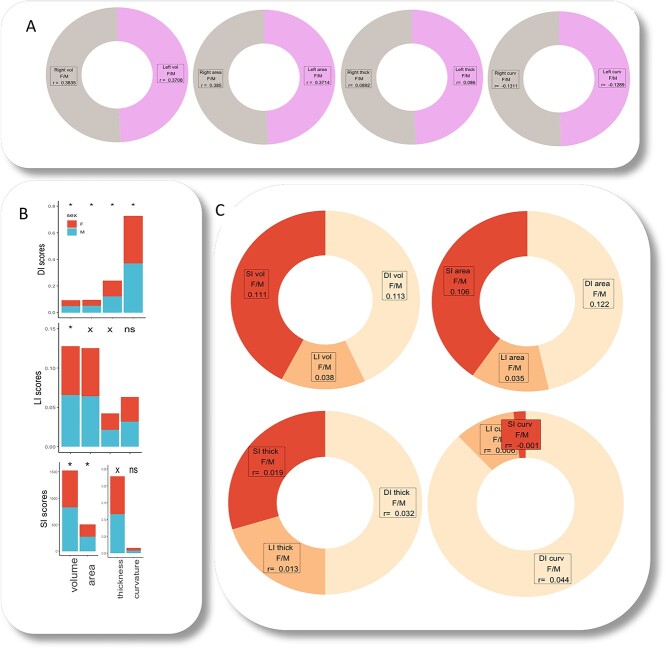
Group analyses and correlations of males/females with index values. Pearson correlation coefficients are given for sex versus raw values in the left and right hemisphere over aggregated regions of interest for all cortical measures—volume, area, thickness, and curvature (A). Significance levels of paired *t*-tests between male and female index values for cortical measures are marked as “^*^,” “x,” and “ns” if less than 0.001, less than 0.05 but higher than 0.001, and higher than 0.05, respectively (B). Pearson correlation coefficients for sex versus each asymmetry index across cortical measures (C).

As seen in Fig. 3(A), correlation coefficients with males/females were similar for the left and the right hemisphere. Paired *t*-tests between them for each cortical measures were not significant, showing that neither hemisphere provides robust cues for binary classification. Figure 3(B) summarizes whole brain averages for males and females across indices for surface area, cortical volume, thickness, and mean curvature. Overall, index values in these group analyses were higher for males than for females. For DI, *P*-values were highly significant (under 0.001) across all cortical measures. For LI, *P*-values were highly significant only for volume, they were significant (under 0.05 but over 0.001) for area and thickness, and non-significant for mean curvature. For SI, *P*-values were highly significant for volume and area, significant for thickness, and non-significant for mean curvature.

The analyses contrasting males and females revealed that DI yielded the best outcomes, followed by SI, and then by LI. These results are confirmed by correlation analyses, as seen in Fig. 3(C), which presents an informal comparison of Pearson correlation coefficients for each asymmetry index and males/females across cortical measures. Higher values indicate better classification performance. Overall, DI (light orange) has associated the highest coefficients, especially for mean curvature, with SI (mid-orange) a good second, especially for average volume and surface area. Of the three, LI (deep orange) yielded the lowest coefficients and therefore the poorest classification.

## Discussion

We provided evidence that brain asymmetry is globally different in males and females, with the distance index being the most suitable methodological tool for evaluating these differences. Machine learning and robustness analyses contrasted the distance index with the laterality index and the subtraction index, which are based on local pairwise differences between brain regions, in their ability to distinguish males from females. Random Forest models performed overall better in terms of accuracy, non-randomness, and *Cohen’s Kappa* for DI volume, area, and curvature compared with SI and especially to LI, which fared poorly. Interestingly, binary classification failed for cortical thickness across indices, suggesting the existence of regional differences in thickness that cannot be captured by whole-brain analyses.

For each index, *t*-test results between males and females were significant, especially for the distance index. Moreover, unlike DI values, LI and SI values did not differ significantly in males and females for mean curvature—incidentally, DI correlation coefficients with males/females were highest for mean curvature. When investigating index robustness in upper and lower subsamples corresponding to highest and lowest image-quality metrics, DI yielded the highest percentage of significant *t*-tests (80%) in group analyses of males versus females. LI scores were significant in only 25% of the tests, and SI scores were not significant. Moreover, informal correlation analyses between sex and index measures illustrated in Fig. 3(C) underscore the robustness of DI as a global coherence index for encoding sexual dimorphism.

When looking at raw cortical measures, correlation coefficients with sex did not differ between the left and the right hemisphere, suggesting that neither of them alone encodes cues for binary classification. In contrast, it is the combined magnitude of proportions between regions across hemispheres that appears to be a useful metric, which suggests that specific growth rules are at work ensuring cortical asymmetry differences in males and females. The fact that DI was a better biomarker of sexual dimorphism compared with LI or SI suggests that individual ROIs have different target sizes in non-random ways that is, they must be proportional to ROIs in the opposite hemisphere, which highlights a complex pattern of contralateral growth regulations across left and right side (Genikhovich and Technau 2017). In other words, asymmetry must have evolved in humans not as weighted pairwise differences between the left and the right hemisphere, which would simply involve scaling, but as globally coherent relationships relying on hemisphere target size, interactions among ROIs within each hemisphere, and inherent positional identity of each region to ensure effective signaling between ipsilateral and contralateral regions.

In the early mammalian cortex, neurogenesis ensures the growth of neural precursor cells (Eriksson et al. 1998) via contralateral signaling. Importantly, neurogenesis continues to ensure plasticity of selected regions in the adult brain (for reviews, see Kolb and Whishaw 1989; Plowman and Kleim 2010), especially following injury (Shohayeb 2018). Indeed, extensive changes in adult animals occur after brain damage that highlights the plasticity of uninjured networks. When stimulated, injury ipsilateral regions elicited both ipsilateral and contralateral motor responses (Murphy and Corbett 2009; Ueno et al. 2012), underscoring the importance of a continuous dialog between hemispheres. An important issue is whether contralateral signaling and neurogenesis are dependent on the properties of the corpus callosum, which links the two hemispheres. Early studies support the hypothesis that callosal axonal fibers mostly connect homotopic cortical areas, yet recent studies provide evidence that interhemispheric callosal connections are heterotopic for the most part, and are strongly involved in brain networks across hemispheres (Swanson et al. 2017; Szczupak et al. 2022). Even more remarkably, interhemispheric connectivity was found in mammals with or without a corpus callosum, which helps define a timeline of interhemispheric communications such that a gray matter connectome must precede callosal growth (Suarez et al. 2018). Nevertheless, abnormalities of gray as well as white matter have been reported in neurodevelopmental disorders including schizophrenia (Bilder et al. 1994; Honea et al. 2005; Steinmann et al. 2021), autism (Hazlett et al. 2005; Herbert et al. 2005), stuttering (Foundas et al. 2003), and Tourette syndrome (Hong et al. 2002), supporting a key role for the callosum in brain function. These findings lay bare the field of future conjectures on the origins of brain asymmetry, which develops both via signaling and across white matter bundles in the corpus callosum through global reorganization instead of pairwise connections across hemispheres.

Sexual dimorphism in the structure of gray and white matter is well documented in literature. Cerebral asymmetry patterns in neonates are opposite to that of older children and adults, suggesting that adult patterns arise before birth and persist throughout postnatal brain development, driven by genetics as well by experience (Gilmore et al. 2007). Cortical asymmetry can be observed as early as 22 weeks of gestation (Chi et al. 1977; Hering-Hanit et al. 2001), being more leftward in males (Kivilevitch et al. 2010) and predominantly rightward in premature males and females (Dubois et al. 2008; Lin et al. 2012). In the adult brain, asymmetries include the right hemisphere being larger than the left, mainly due to more white matter on the right, and fronto-occipital asymmetry with larger right prefrontal cortex compared with the left and larger left occipital cortex compared with the right (Nopoulos et al. 2000; Toga and Thompson 2003). White matter continues to increase in volume throughout life (Lenroot et al. 2007; Giedd et al. 1999; Sowell et al. 2002), but gray matter reaches a peak around 10 years of age, slightly earlier in females for temporal and frontal lobes (Tanaka et al. 2012). Although males have larger brain volumes and surface areas compared with females, cortical thickness is greater in females (Ritchie et al. 2018).

There is greater variance in males compared with females for volume and surface area, suggesting that individual differences are more important in males (Giedd et al. 1996). However, asymmetry patterns appear to be systematic and predictable rather than random, as witness good DI performance, which suggests that mere variance or individual differences cannot explain away the sexual dimorphism of cortical asymmetries. Growth rates triggered by genetic and hormonal factors are more likely candidates, as they impact cortical growth across the lifespan (^60,51^; Sisk and Zehr 2005), have associated effects on cognition and mental illness, and account for increased variability of male brains via genomic imprinting (Dulac and Christopher 2013), mitochondrial DNA mutations passed on maternally (Gemmell et al. 2004), X chromosome effects (Craig et al. 2009)), and further mechanisms protecting females from damaging consequences of genetic mutations (Robinson et al. 2013). Moreover, testosterone and/or estradiol in males increase the number of calcium-binding proteins, which are known to prevent neuronal death, thus resulting in males having a greater number of neurons than females (Segovia et al. 1999).

To summarize, we found DI to be a robust biomarker of cortical asymmetry. Of the three indices, it captured best whole brain differences between the sexes, with female brains being structurally more coherent than male brains. Importantly, the whole brain size may remain unchanged, while degree of coherence may differ. Also, reduced lateralization does not entail higher coherence, and, conversely, highly lateralized structures need not be less coherent, since an interaction among ROIs within each hemisphere may or may not occur. Sexual dimorphism in brain structure appears to draw on the rules governing form and proportion, which are fundamental for the development of biological organisms (Harris et al. 2021). However, there are currently no advanced reports on the impact of genetic, epigenetic, and hormonal factors on distinct types of brain asymmetry in males versus females. Moreover, our findings cannot distinguish the effects of developmental processes from the effects of genetic hardwiring of proportion parameters as they converge toward global differences in brain asymmetry between young adult males and females. Further studies are needed, in the future, to explore the biological basis of various types of asymmetries in the human brain.

## CRediT statement

M.D. was responsible for manuscript conceptualization, formal analysis, visualization, writing, and editing.

## References

[2] Atkinson D , Hill DL, Stoyle PN, Summers PE, Keevil SF. Automatic correction of motion artifacts in magnetic resonance images using an entropy focus criterion. IEEE Trans Med Imaging. 1997:16(6):903–910. 9533590 10.1109/42.650886

[3] Avants BB , Epstein CL, Grossman M, Gee JC. Symmetric diffeomorphic image registration with cross-correlation: evaluating automated labeling of elderly and neurodegenerative brain. Med Image Anal. 2008:12(1):26–41. 17659998 10.1016/j.media.2007.06.004 PMC2276735

[4] Bilder RM , Wu H, Bogerts B, Degreef G, Ashtari M, Alvir JM, Snyder PJ, Lieberman JA. Absence of regional hemispheric volume asymmetries in first-episode schizophrenia. Am J Psychiatry. 1994:151(10):1437–1447. 8092337 10.1176/ajp.151.10.1437

[5] Boersma B , Wit JM. Catch-up growth. Endocr Rev. 1997:18(5):646–661. 9331546 10.1210/edrv.18.5.0313

[6] Breiman L . Random Forests. Mach Learn. 2001:45(1):5–32.

[7] Brunoni AR , Moffa AH, Fregni F, Palm U, Padberg F, Blumberger DM, Daskalakis ZJ, Bennabi D, Haffen E, Alonzo A, et al. Transcranial direct current stimulation for acute major depressive episodes: meta-analysis of individual patient data. BJPsych. 2016:208(6):522–531. 27056623 10.1192/bjp.bp.115.164715 PMC4887722

[8] Busse SM , McMillen PT, Levin M. Cross-limb communication during Xenopus hindlimb regenerative response: non-local bioelectric injury signals. Development. 2018:145(19):dev164210. 30126906 10.1242/dev.164210

[9] Chi JG , Dooling EC, Gilles FH. Left-right asymmetries of the temporal speech areas of the human fetus. Arch Neurol. 1977:34(6):346–348. 860936 10.1001/archneur.1977.00500180040008

[10] Chiarello C , Vazquez D, Felton A, McDowell A. Structural asymmetry of the human cerebral cortex: regional and between-subject variability of surface area, cortical thickness, and local gyrification. Neuropsychologia. 2016:93:365–379. 26792368 10.1016/j.neuropsychologia.2016.01.012 PMC4947520

[11] Craig IW , Haworth CM, Plomin R. Commentary on “a role for the X chromosome in sex differences in variability in general intelligence?” (Johnson et al. 2009). Perspect Psychol Sci. 2009:4(6):615–621. 26161737 10.1111/j.1745-6924.2009.01170.x

[12] Dale AM , Fischl B, Sereno MI. Cortical surface-based analysis: I. Segmentation and surface reconstruction. NeuroImage. 1999:9(2):179–194. 9931268 10.1006/nimg.1998.0395

[13] De Courten-Myers G . The human cerebral cortex: gender differences in structure and function. J Neuropathol Exp Neurol. 1999:58(3):217–226. 10197813 10.1097/00005072-199903000-00001

[14] Debat V , Peronnet F. Asymmetric flies: the control of developmental noise in drosophila. Fly. 2013:7(2):70–77. 23519089 10.4161/fly.23558 PMC3732334

[15] Delgado R , Tibau X-A. Why Cohen’s *Kappa* should be avoided as performance measure in classification. PLoS One. 2019:14(9):e0222916. 31557204 10.1371/journal.pone.0222916 PMC6762152

[16] Desikan RS , Ségonne F, Fischl B, Quinn BT, Dickerson BC, Blacker D, Buckner RL, Dale AM, Maguire RP, Hyman BT, et al. An automated labeling system for subdividing the human cerebral cortex on MRI scans into gyral based regions of interest. NeuroImage. 2006:31(3):968–980. 16530430 10.1016/j.neuroimage.2006.01.021

[17] Di Martino A , Yan CG, Li Q, Denio E, Castellanos FX, Alaerts K, Anderson JS. The autism brain imaging data exchange: towards a large-scale evaluation of the intrinsic brain architecture in autism. Mol Psychiatry. 2014:19(6):659–667. 23774715 10.1038/mp.2013.78 PMC4162310

[18] Dubois J , Benders M, Cachia A, Lazeyras F, Ha-Vinh Leuchter R, Sizonenko SV, Borradori-Tolsa C, Mangin JF, Huppi PS. Mapping the early cortical folding process in the preterm newborn brain. Cereb Cortex. 2008:18(6):1444–1454. 17934189 10.1093/cercor/bhm180

[19] Dulac C , Christopher G. Genomic imprinting in the adult and developing brain. In: Pfaff D, Christen Y, editors. Multiple origins of sex differences in brain: research and perspectives in endocrine interactions. Berlin: Springer; 2013. pp. 35–41

[20] Eriksson PS , Perfilieva E, Bjork-Eriksson T, Alborn A-M, Nordborg C, Peterson DA, Gage FH. Neurogenesis in the adult human hippocampus. Nat Med. 1998:4(11):1313–1317. 9809557 10.1038/3305

[21] Esteban O , Birman D, Schaer M, Koyejo OO, Poldrack RA, Gorgolewski KJ. MRIQC: advancing the automatic prediction of image quality in MRI from unseen sites. PLoS One. 2017:2017(9):12. 10.1371/journal.pone.0184661 PMC5612458 28945803

[22] Esteves M , Moreira PS, Marques P, Castanho TC, Magalhães R, Amorim L, Portugal-Nunes C, Soares JM, Coelho A, Almeida A, et al. Asymmetrical subcortical plasticity entails cognitive progression in older individuals. Aging Cell. 2019:18(1):e12857. 30578611 10.1111/acel.12857 PMC6351824

[23] Fischerauer EE , Manninger M, Seles M, Janezic G, Pichler K, Ebner B, Weinberg AM. BMP-6 and BMPR-1a are up-regulated in the growth plate of the fractured tibia. J Orthop Res. 2013:31(3):357–363. 23097200 10.1002/jor.22238

[24] Fleiss JL . Statistical methods for rates and proportions. 2nd ed. New York: John Wiley; 1981.

[99] Fonov VS , Evans AC, McKinstry RC, Almli CR, Collins DL. Unbiased nonlinear average age-appropriate brain templates from birth to adulthood. Neuroimage. 2009:47:S102.

[25] Foundas AL , Corey DM, Angeles V, Bollich AM, Crabtree-Hartman E, Heilman KM. Atypical cerebral laterality in adults with persistent developmental stuttering. Neurology. 2003:61(10):1378–1385. 14638959 10.1212/01.wnl.0000094320.44334.86

[26] Ganzetti M , Wenderoth N, Mantini D. Intensity inhomogeneity correction of structural MR images: a data-driven approach to define input algorithm parameters. Front Neuroinform. 2016a:10:10. 27014050 10.3389/fninf.2016.00010 PMC4791378

[28] Gemmell NJ , Metcalf VJ, Allendorf FW. Mother’s curse: the effect of mtDNA on individual fitness and population viability. Trends in Ecol Evol. 2004:19(5):238–244. 10.1016/j.tree.2004.02.002 16701262

[29] Genikhovich G , Technau U. On the evolution of bilaterality. Development. 2017:144(19):3392–3404. 28974637 10.1242/dev.141507

[30] Giedd JN , Vaituzis AC, Hamburger SD, Lange N, Rajapakse JC, Kaysen D, Vauss YC, Rapoport JL. Quantitative MRI of the temporal lobe, amygdala, and hippocampus in normal human development: ages 4–18 years. J Comp Neurol. 1996:366(2):223–230. 8698883 10.1002/(SICI)1096-9861(19960304)366:2<223::AID-CNE3>3.0.CO;2-7

[31] Giedd JN , Blumenthal J, Jeffries NO, Castel FX, Liu H, Zijdenbos A, Paus T, Evans AC, Rapoport JL. Brain development during childhood and adolescence: a longitudinal MRI study. Nat Neurosci. 1999:2(10):861–863. 10491603 10.1038/13158

[32] Gilmore JH , Lin W, Prastawa MW, Looney CB, Sampath Y, Vetsa K, Gerig G. Regional gray matter growth, sexual dimorphism, and cerebral asymmetry in the neonatal brain. J Neurosci. 2007:27(6):1255–1260. 17287499 10.1523/JNEUROSCI.3339-06.2007 PMC2886661

[33] Gomez J , Zhen Z, Weiner KS. Human visual cortex is organized along two genetically opposed hierarchical gradients with unique developmental and evolutionary origins. PLoS Biol. 2019:17(7):e3000362. 31269028 10.1371/journal.pbio.3000362 PMC6634416

[34] Good CD , Johnsrude IS, Ashburner J, Henson RNA, Friston KJ, Frackowiak RSJ. A voxel-based morphometric study of ageing in 465 normal adult human brains. NeuroImage. 2001:14(1 Pt 1):21–36. 11525331 10.1006/nimg.2001.0786

[35] Grimes DT . Developmental biology: go with the flow to keep the body straight. Curr Biol. 2019:29(3):R101–R103. 30721676 10.1016/j.cub.2018.12.011

[36] Guadalupe T , Zwiers MP, Wittfeld K, Teumer A, Vasquez AA, Hoogman M, Hagoort P, Fernandez G, Buitelaar J, van Bokhoven H, et al. Asymmetry within and around the human planum temporale is sexually dimorphic and influenced by genes involved in steroid hormone receptor activity. Cortex. 2015:62:41–55. 25239853 10.1016/j.cortex.2014.07.015

[37] Guo JY , Isohanni M, Miettunen J, Jaaskelainen E, Kiviniemi V, Nikkinen J, Remes J, Huhtaniska S, Veijola J, Jones PB, et al. Brain structural changes in women and men during midlife. Neurosci Lett. 2016:615:107–112. 26777626 10.1016/j.neulet.2016.01.007 PMC4762229

[38] Habib R , Nyberg L, Tulving E. Hemispheric asymmetries of memory: the HERA model revisited. Trends Cogn Sci. 2003:7(6):241–245. 12804689 10.1016/s1364-6613(03)00110-4

[39] Harris MP , Daane JM, Lanni J. Through veiled mirrors: fish fins giving insight into size regulation. WIREs Dev Biol. 2021:10(4):e381. 10.1002/wdev.381 32323915

[40] Hazlett HC , Poe M, Gerig G, Smith RG, Provenzale J, Ross A, Gilmore J, Piven J. Magnetic resonance imaging and head circumference study of brain size in autism: birth through age 2 years. Arch Gen Psychiatry. 2005:62(12):1366–1376. 16330725 10.1001/archpsyc.62.12.1366

[41] Herbert MR , Ziegler DA, Deutsch CK, O’Brien LM, Kennedy DN, Filipek PA, Bakardjiev AI, Hodgson J, Takeoka M, Makris N, et al. Brain asymmetries in autism and developmental language disorder: a nested whole-brain analysis. Brain. 2005:128(Pt 1):213–226. 15563515 10.1093/brain/awh330

[42] Hering-Hanit R , Achiron R, Lipitz S, Achiron A. Asymmetry of fetal cerebral hemispheres: in utero ultrasound study. Arch Dis Child Fetal Neonatal Ed. 2001:85(3):f194–f196. 11668162 10.1136/fn.85.3.F194 PMC1721319

[43] Honea R , Crow TJ, Passingham D, Mackay CE. Regional deficits in brain volume in schizophrenia: a meta-analysis of voxel-based morphometry studies. Am J Psychiatry. 2005:162(12):2233–2245. 16330585 10.1176/appi.ajp.162.12.2233

[44] Hong KE , Ock SM, Kang MH, Kim CE, Bae JN, Lim MK, Suh CH, Chung SJ, Cho SC, Lee JS. The segmented regional volumes of the cerebrum and cerebellum in boys with Tourette syndrome. J Korean Med Sci. 2002:17(4):530–536. 12172051 10.3346/jkms.2002.17.4.530 PMC3054894

[46] Jansen A , Menke R, Sommer J, Forster AF, Bruchmann S, Hempleman J, Weber B, Knecht S. The assessment of hemispheric lateralization in functional MRI – robustness and reproducibility. NeuroImage. 2006:33(1):204–217. 16904913 10.1016/j.neuroimage.2006.06.019

[97] Jeyaraman BP , Olsen LR, Wambugu M. Practical machine learning with R: Define, build, and evaluate. Machine learning models for real-world applications. Packt Publishing; 2019.

[48] Kivilevitch Z , Achiron R, Zalel Y. Fetal brain asymmetry: in utero sonographic study of normal fetuses. Am J Obstet Gynecol. 2010:202(359):e1–e8. 10.1016/j.ajog.2009.11.001 20074689

[49] Klein A , Ghosh SS, Bao FS, Giard J, Häme Y, Stavsky E. Mindboggling morphometry of human brains. PLoS Comput Biol 2017:13(2), 2, e1005350. 28231282 10.1371/journal.pcbi.1005350 PMC5322885

[50] Kolb B , Whishaw IQ. Plasticity in the neocortex: mechanisms underlying recovery from early brain damage. Prog Neurobiol. 1989:32(4):235–276. 2655008 10.1016/0301-0082(89)90023-3

[101] Kong X-Z , Mathias SR, Guadalupe T, ENIGMA laterality working group, Glahn DC, Franke B, Crivello F, Tzourio-Mazoyer N, Fisher SE, Thompson PM, et al. Mapping cotical brain asymmetry in 17,141 healthy individuals worldwide via de ENIGMA Consortium. PNAS. 2018:115(22):5154–E5153. 10.1073/pnas.1718418115 PMC5984496 29764998

[51] Korol DL . Role of estrogen in balancing contributions from multiple memory systems. Neurobiol Learn Mem. 2004:82(3):309–323. 15464412 10.1016/j.nlm.2004.07.006

[52] Kuhn M . Building predictive models in R using the caret package. J Stat Softw. 2008:28(5):1–26.27774042

[54] Lander AD . Pattern, growth, and control. Cell. 2011:144(6):955–969. 21414486 10.1016/j.cell.2011.03.009 PMC3128888

[55] Lehtola SJ , Tuulari JJ, Karlsson L, Parkkola R, Merisaari H, Saunavaara J, Lahdesmaki T, Scheinin NM, Karlsson H. Associations of age and sex with brain volumes and asymmetry in 2-5-week-old infants. Brain Struct Funct. 2019:224(1):501–513. 30390153 10.1007/s00429-018-1787-x PMC6373364

[56] Lenroot RK , Gogtay N, Greenstein DK, Wells EM, Wallace GL, Clasen LS, Blumenthal JD, Lerch J, Zijdenbos AP, Evans AC, et al. Sexual dimorphism of brain developmental trajectories during childhood and adolescence. NeuroImage. 2007:36(4):1065–1073. 17513132 10.1016/j.neuroimage.2007.03.053 PMC2040300

[58] Lin PY , Roche-Labarbe N, Dehaes M, Fenoglio A, Grant PE, Franceschini MA. Regional and hemispheric asymmetries of cerebral hemodynamic and oxygen metabolism in newborns. Biomed Opt Biomed. 2012:23(2):339–348. 10.1093/cercor/bhs023 PMC3584954 22328446

[59] Lotze M , Domin M, Gerlach FH, Gaser C, Lueders E, Schmidt CO, Neumann N. Novel findings from 2838 adult brains on sex differences in Gray matter brain volume. Sci Rep. 2019:9(1):1671. 30737437 10.1038/s41598-018-38239-2 PMC6368548

[60] McEwen BS , Milner TA. Understanding the broad influence of sex hormones and sex differences in the brain. J Neurosci Res. 2017:95(1–2):24–39. 27870427 10.1002/jnr.23809 PMC5120618

[61] Mowinckel AM , Vidal-Piñeiro D. Visualisation of brain statistics with R-packages ggseg and ggseg3d. Advances in Methods and Practices in Psychological Science (AMPPS). 2019:3(4):s.466–483.

[62] Murphy TH , Corbett D. Plasticity during stroke recovery: from synapse to behaviour. Nat Rev Neurosci. 2009:10(12):861–872. 19888284 10.1038/nrn2735

[63] Nopoulos P , Flaum M, O’Leary D, Andreasen NC. Sexual dimorphism in the human brain: evaluation of tissue volume, tissue composition and surface anatomy using magnetic resonance imaging. Psychiatry Res. 2000:98(1):1–13. 10708922 10.1016/s0925-4927(99)00044-x

[65] Plowman EK , Kleim JA. Motor cortex reorganization across the lifespan. J Commun Disord. 2010:43(4):286–294. 20478572 10.1016/j.jcomdis.2010.04.005

[66] Power JD , Barnes KA, Snyder AZ, Schlaggar BL, Petersen SE. Spurious but systematic correlations in functional connectivity MRI networks arise from subject motion. NeuroImage. 2012:59(3):2142–2154. 22019881 10.1016/j.neuroimage.2011.10.018 PMC3254728

[67] Prader A , Tanner JM, von Harnack G. Catch-up growth following illness or starvation. An example of developmental canalization in man. J Pediatr. 1963:62(5):646–659. 13985887 10.1016/s0022-3476(63)80035-9

[68] R Core Team , 2012. R: a language and environment for statistical computing. Vienna: R Foundation for Statistical Computing. http://www.r-project.org/.

[69] Rajagopalan V , Scott J, Habas PA, Kim K, Rousseau F, Glenn OA, Studholme C. Mapping directionality specific volume changes using tensor-based morphometry: an application to the study of gyrogenesis and lateralization of the human fetal brain. NeuroImage. 2011:63(2):947–958. 10.1016/j.neuroimage.2012.03.092 PMC3732053 22503938

[70] Rao CV , Wolf DM, Arkin AP. Control, exploitation and tolerance of intracellular noise. Nature. 2002:420(6912):231–237. 12432408 10.1038/nature01258

[72] Ritchie SJ , Cox SR, Shen X, Lombardo MV, Reus LM, Alloza C, Harris MA, Alderson HL, Hunter S, Neilson E, et al. Sex differences in the adult human brain: evidence from 5216 UK Biobank participants. Cereb Cortex. 2018:28(8):2959–2975. 29771288 10.1093/cercor/bhy109 PMC6041980

[73] Roalf DR , Quarmley M, Elliott MA, Satterthwaite TD, Vandekar SN, Ruparel K, Gennatas ED, Calkins ME, Moore TM, Hopson R, et al. The impact of quality assurance assessment on diffusion tensor imaging outcomes in a large-scale population-based cohort. NeuroImage. 2016:125:903–919. 26520775 10.1016/j.neuroimage.2015.10.068 PMC4753778

[74] Robinson EB , Lichtenstein P, Anckarsäter H, Happé F, Ronald A. Examining and interpreting the female protective effect against autistic behavior. PNAS. 2013:110(13):5258–5262. 23431162 10.1073/pnas.1211070110 PMC3612665

[75] Roselló-Dıéz A , Madisen L, Bastide S, Zeng H, Joyner AL. Cell-nonautonomous local and systemic responses to cell arrest enable long-bone catch-up growth in developing mice. PLoS Biol. 2018:16(6):e2005086. 29944650 10.1371/journal.pbio.2005086 PMC6019387

[76] Rosen AFG , Roalf DR, Ruparel K, Blake J, Seelaus K, Villa LP, Ciric R, Cook PA, Davatzikos C, Elliott MA, et al. Quantitative assessment of structural image quality. NeuroImage. 2018:169:407–416. 29278774 10.1016/j.neuroimage.2017.12.059 PMC5856621

[77] Ruigrok ANV , Salimi-Khorshidi G, Lai M-L, Baron-Cohen S, Lombardo MV, Tait RJ, Suckling J. A meta-analysis of sex differences in human brain structure. Neurosci Biobehav Rev. 2014:39(100):34–50. 24374381 10.1016/j.neubiorev.2013.12.004 PMC3969295

[78] Sanchis-Segura C , Ibañez-Gual MV, Adrián-Ventura J, Aguirre N, Gómez-Cruz ÁJ, Avila C, Forn C. Sex differences in gray matter volume: how many and how large are they really? Biol Sex Differ. 2019:10(32):1–19. 31262342 10.1186/s13293-019-0245-7 PMC6604149

[80] Segovia S , Guillamon A, del Cerro MCR, Ortega E, Perez-Laso C, Rodriguez-Zafra M, Beyer C. The development of brain sex differences: a multisignaling process. Behav Brain Res. 1999:1999(105):69–80. 10.1016/s0166-4328(99)00083-2 10553691

[81] Shapleske J , Rossell SL, Woodruff PWR, David AS. The planum temporale: a systematic, quantitative review of its structural, functional and clinical significance. Brain Res Rev. 1999:29(1):26–49. 9974150 10.1016/s0165-0173(98)00047-2

[82] Shohayeb B , Diab M, Ahmed M, Ng DCH. Factors that influence adult neurogenesis as potential therapy. Transl Neurodegener. 2018:7(4):4. 29484176 10.1186/s40035-018-0109-9 PMC5822640

[83] Sisk CL , Zehr JL. Pubertal hormones organize the adolescent brain and behavior. Front Neuroendocrinol. 2005:26(3–4):163–174. 16309736 10.1016/j.yfrne.2005.10.003

[84] Snoek L , Van der Miesen MM, Beemsterboer T, Van der Leij A, Eigenhuis A, Scholte HS. The Amsterdam open MRI collection, a set of multimodal MRI datasets for individual difference analyses. Sci Data. 2021:8:85. 10.1038/s41597-021-00870-6 PMC7979787 33741990

[85] Sowell ER , Trauner DA, Gamst A, Jernigan TL. Development of cortical and subcortical brain structures in childhood and adolescence: a structural MRI study. Dev Med Child Neurol. 2002:44(01):4–16. 11811649 10.1017/s0012162201001591

[86] Sowell ER , Peterson BS, Kan E, Woods RP, Yoshii J, Bansal R, Xu D, Zhu H, Thompson PM, Toga AW. Sex differences in cortical thickness mapped in 176 healthy individuals between 7 and 87 years of age. Cereb Cortex. 2007:17(7):1550–1560. 16945978 10.1093/cercor/bhl066 PMC2329809

[87] Steinmann S , Lyall AE, Langhein M, Nagele FL, Rauh J, Cetin-Karayumak S, Zhang F, Mussmann M, Billah T, Makris N, et al. Sex-related differences in white matter asymmetry and its implications for verbal working memory in psychosis high-risk state. Front Psychiatry. 2021:12:686967. 34194350 10.3389/fpsyt.2021.686967 PMC8236502

[100] Suarez R , Paolino A, Fenlon LR, Morcom LR, Kozulin P, Kurniawan ND, Richards LJ. A pan-mammalian map of interhemispheric brain connections predates the evolution of the corpus callosum. PNAS. 2018:115(38):9622–9627. 30181276 10.1073/pnas.1808262115 PMC6156618

[88] Swanson LW , Hahn JD, Sporns O. Organizing principles for the cerebral cortex network of commissural and association connections. PNAS. 2017:114(45):E9692–E9701. 29078382 10.1073/pnas.1712928114 PMC5692583

[89] Szczupak D , Meneses Iack P, Rayee D, Liu C, Lent R, Tovar-Moll F, Silva AC. The relevance of heterotopic callosal fibers to interhemispheric connectivity of the mammalian brain. Cereb Cortex. 2022:33(8):1–9. 36178137 10.1093/cercor/bhac377 PMC10110439

[90] Tanaka C , Matsui M, Uematsu A, Noguchi K, Miyawaki T. Developmental trajectories of the fronto-temporal lobes from infancy to early adulthood in healthy individuals. Dev Neurosci. 2012:34(6):477–487. 23257954 10.1159/000345152

[91] Tanner JM . Regulation of growth in size in mammals. Nature. 1963:199(4896):845–850. 14079891 10.1038/199845a0

[92] Toga AW , Thompson PM. Mapping brain asymmetry. Nat Rev Neurosci. 2003:4(1):37–48. 12511860 10.1038/nrn1009

[93] Ueno M , Hayano Y, Nakagawa H, Yamashita T. Intraspinal rewiring of the corticospinal tract requires target-derived brain-derived neurotrophic factor and compensates lost function after brain injury. Brain. 2012:135(4):1253–1267. 22436236 10.1093/brain/aws053

[94] Waddington CH . Canalization of development and genetic assimilation of acquired characters. Nature. 1959:183(4676):1654–1655. 13666847 10.1038/1831654a0

[95] Zhang Y , Brady M, Smith S. Segmentation of brain MR images through a hidden Markov random field model and the expectation-maximization algorithm. IEEE Trans Med Imaging. 2001:20(1):45–57. 11293691 10.1109/42.906424

[96] Zhen Z , Yang Z, Huang L, Kong XZ, Wang X, Dang X, Huang Y, Song Y, Liu J. Quantifying interindividual variability and asymmetry of face-selective regions: a probabilistic functional atlas. NeuroImage. 2015:113:13–25. 25772668 10.1016/j.neuroimage.2015.03.010

[98] Zuo Z , Subgang A, Abaei A, Rottbauer W, Stiller D, Ma G, Rasche V. Assessment of longitudinal reproducibility of mice LV function parameters at 11.7 T derived from self-gated CINE MRI. Biomed Res Int. 2017:8392952:10. 10.1155/2017/8392952 PMC5340939 28321415

